# Corrosion Behavior of 304 Stainless Steel During Three-Year Atmospheric Field Exposure in Antarctica

**DOI:** 10.3390/ma19132754

**Published:** 2026-06-29

**Authors:** Ting Peng, Shicheng Wang, Sizhi Zuojiang, Zihao Tian, Yijing Sun, Xuzhou Jiang, Dongbai Sun

**Affiliations:** 1 School of Materials Science and Engineering & Southern Marine Science and Engineering Guangdong Laboratory (Zhuhai), Sun Yat-sen University, Guangzhou 510006, China; pengt39@mail2.sysu.edu.cn (T.P.); zuojiangsizhi@163.com (S.Z.); tianzh9@mail2.sysu.edu.cn (Z.T.); 2 Guangzhou Customs Technology Center, Guangzhou 510623, China; wscxmu@163.com; 3 Sino-French Institute of Nuclear Engineering, Sun Yat-sen University, Zhuhai 519082, China; 4 Nanotechnology Research Center, Sun Yat-sen University, Guangzhou 510275, China

**Keywords:** 304 stainless steel, Antarctica, atmospheric corrosion, pitting corrosion, passive film

## Abstract

Three-year atmospheric field-exposure tests were conducted on 304 austenitic stainless steel at the Great Wall and Zhongshan Stations in Antarctica to evaluate its corrosion behavior under severe polar conditions. The exposed specimens were dominated by localized corrosion with pronounced pitting characteristics at both sites. Corrosion was more severe at Zhongshan Station, and the mean corrosion rates at Great Wall and Zhongshan Stations were 1.428 and 1.643 μm y^−1^, respectively. The mean/maximum pit depths were 4.16/5.51 μm at Great Wall Station and 5.85/8.24 μm at Zhongshan Station. Raman spectroscopy, X-ray photoelectron spectroscopy (XPS), grazing-incidence X-ray diffraction (GIXRD), and focused ion beam-transmission electron microscopy (FIB-TEM) showed that the corrosion products consisted mainly of β-FeOOH, α-FeOOH, and γ-Fe_2_O_3_, and the Antarctic exposure substantially altered the thickness, structure, and electrochemical response of the passive film. Compared with the unexposed specimen, the exposed specimens exhibited markedly lower charge-transfer resistance and higher donor density, indicating degradation of the protective passive film. Combined with the site-specific environmental features, the lower temperature, more intense freeze–thaw cycling, freezing-induced concentration of electrolytes, and stronger irradiation at Zhongshan Station are inferred to promote Cl^−^ enrichment in localized surface liquid films and destabilization of the passive film, thereby accelerating pit initiation and growth. These findings provide a mechanistic basis for material selection and corrosion-protection design for 304 stainless steel in polar engineering environments.

## 1. Introduction

304 stainless steel combines favorable mechanical properties, corrosion resistance, weldability and processability, and is therefore widely used in outdoor polar infrastructure, buildings, aerospace systems, transportation and petrochemical facilities [1]. In atmospheric environments, a compact passive film forms spontaneously on the surface, imparting high corrosion resistance [2]. Even in the presence of a thin electrolyte film, this passive layer is generally resistant to damage and can rapidly self-repair once locally disrupted.

Under aggressive service conditions, especially marine atmospheres, the situation is markedly different. Wind-borne chloride-containing sea-salt aerosols deposit on the steel surface; after deliquescence, released chloride ions readily induce breakdown of the passive film and initiate severe localized corrosion, particularly pitting [3]. Increasing chloride concentration lowers the pitting potential and therefore increases the susceptibility to pit initiation. Extensive studies have shown that stainless steel components exposed in marine atmospheres undergo significant corrosion, whereas those in inland urban atmospheres often show no obvious corrosion traces [4]. Environmental factors such as chloride deposition, relative humidity, temperature, and pressure strongly influence the corrosion behavior of stainless steels, and chloride is especially aggressive in hot and humid marine atmospheres [5]. For example, 304 stainless steel in high-temperature, high-humidity salt-spray environments typically undergoes pitting corrosion, with β-FeOOH as a dominant corrosion product and a passive film progressively losing stability with exposure time.

The Antarctic atmosphere is characterized by persistent low temperatures, strong winds, high salt deposition, frequent wet–dry transition, intense freeze–thaw cycling, and intense ultraviolet radiation. The coupling of these factors can impose distinctive effects on electrochemical corrosion, passive-film stability, and corrosion-product evolution on stainless-steel surfaces [6]. Previous studies have shown that these environmental factors can influence atmospheric corrosion by altering surface electrolyte formation, passive-film stability, oxygen transport and corrosion-product evolution [7]. However, most available studies have focused either on reference metals in polar atmospheres or on stainless steels under marine atmospheric and laboratory-simulated conditions. The corrosion evolution of 304 stainless steel during long-term exposure at Antarctic stations remains insufficiently understood, particularly regarding how transient thin liquid films formed during freezing–thawing cycles contribute to the initiation and propagation of pitting corrosion under site-specific polar conditions. Therefore, a systematic investigation based on actual field exposure at different Antarctic stations is still required.

Recently, Sun et al. [8] investigated the combined effects of low temperature and accelerated seawater corrosion on the mechanical properties of 304 stainless steel in a polar environment. Their results showed that low temperature increased tensile strength but reduced ductility, localized corrosion decreased both strength and ductility, and a coupling effect between low temperature and corrosion became apparent under severe localized corrosion. In contrast to that accelerated corrosion and mechanical-performance study, the present work is based on three-year atmospheric field exposure at Antarctic stations and focuses on site-specific pit evolution, corrosion-product chemistry, passive-film reconstruction, and electrochemical degradation. Therefore, the present results complement the strength-prediction model of Sun et al. [8] by providing field-exposure corrosion data and mechanistic evidence for 304 stainless steel under real Antarctic atmospheric conditions.

Here, three-year atmospheric field-exposure tests were performed at Great Wall Station and Zhongshan Station in Antarctica. White-light interferometry, scanning electron microscopy (SEM), Raman spectroscopy, GIXRD, FIB-TEM, and electrochemical measurements were combined to systematically investigate the corrosion behavior and mechanism of 304 stainless steel in the Antarctic atmosphere. The study focuses on pit initiation and propagation, changes in passive-film structure and performance, and their links to environmental factors, with the aim of clarifying the corrosion processes of 304 stainless steel under harsh polar atmospheric conditions. The results provide data support and mechanistic insight for material selection, lifetime assessment, and protection design of polar engineering equipment, while also extending the fundamental understanding of stainless-steel corrosion in extreme environments.

## 2. Experimental Methods

### 2.1. Field Exposure Tests at Antarctic Great Wall and Zhongshan Stations

The field exposure experiments were conducted at the Great Wall Station and Zhongshan Station, two Chinese Antarctic research stations. The Great Wall Station is located on King George Island in West Antarctica, with geographical coordinates of approximately 62°13′ S, 58°58′ W, whereas Zhongshan Station is situated in the Larsemann Hills of East Antarctica, at approximately 69°22′ S, 76°22′ E [9]. During the field exposure tests, meteorological parameters, including temperature, relative humidity and solar irradiance, were recorded at 1 h intervals using the meteorological monitoring systems installed at the research stations. The collected data were used to evaluate the environmental characteristics of the two exposure sites during the test period. The exposure experiment was designed as a passive field-exposure test. During the three-year exposure period, the specimens were not manually cleaned, snow or ice was not mechanically scraped from the specimen surfaces, and no maintenance operation involved direct contact with the exposed metal surfaces. Routine checks were limited to visual inspection of the exposure racks and collection of meteorological records. After retrieval, the coupons were handled by their edges or fixture regions, sealed in clean bags, packaged, and transported to the laboratory for subsequent standardized cleaning, weighing, and characterization.

### 2.2. Materials and Specimen Preparation

Stainless steel sheets supplied by Shenzhen Quanfu Metal Co., Ltd. (Shenzhen, China) were used for the outdoor atmospheric exposure tests. The specimens were prepared with dimensions of 150 mm × 100 mm × 3 mm, and four replicate coupons were installed at each exposure site. No surface heat treatment was applied before exposure. A pre-exposure elemental analysis was performed on the as-received coupons using a portable X-ray fluorescence spectrometer (TRACER 5i, Bruker Corporation, Billerica, MA, USA); only the alloying elements identified in the quantitative spectrum are listed in Table 1. The measured composition was essentially consistent with the chemical requirements for Type 304 stainless steel specified for stainless-steel plate, sheet, and strip in ASTM A240/A240M [10]. Specifically, the measured Cr and Ni contents were within or very close to the specified ranges, while the Mn, Si, P, and S contents were below the specified upper limits. Because portable XRF cannot reliably quantify light elements such as C and N, these elements were not included in the XRF-based composition table. Before exposure, specimen cleaning and initial weighing were conducted according to the ISO 8407 cleaning-and-weighing workflow [11]. Briefly, the specimens were sequentially ultrasonically degreased in acetone and anhydrous ethanol for 10 min each, rinsed with deionized water, dried with oil-free air, stored in a desiccator for 24 h, and weighed three times using a balance with an accuracy of 0.01 g; the average value was recorded as the initial mass. Specimens exposed at Great Wall Station were denoted as 304 GW, whereas those exposed at Zhongshan Station were denoted as 304 ZS. Specimens of the same grade stored in a desiccator were used as unexposed references and labeled 304 Blank. After retrieval, the exposed specimens were rinsed with deionized water and anhydrous ethanol to remove soluble salts and loose surface deposits. For the mass-loss calculation, residual corrosion products were removed according to GB/T 16545-2025 by immersion in a 20% nitric acid solution at room temperature for 60 min, followed by sequential rinsing with deionized water and ethanol, drying, and weighing [12]. An unexposed blank coupon was cleaned in parallel to correct for possible cleaning-induced substrate dissolution. Macroscopic corrosion morphology was recorded using a digital camera (Sony Alpha 7C, Sony Corporation, Tokyo, Japan).

### 2.3. Characterization

Surface images, three-dimensional surface morphologies, and cross-sectional profiles were acquired using a three-dimensional optical profilometer (ContourX-200, Bruker Scientific LLC, Billerica, MA, USA). The surface micro-morphologies of the specimens were examined by a scanning electron microscope (Axia ChemiSEM HiVac, Thermo Fisher Scientific, Waltham, MA, USA). Raman spectra were collected using a Raman spectrometer (inVia Reflex, Renishaw plc, Wotton-under-Edge, Gloucestershire, UK) with a 633 nm laser excitation source over the spectral range of 100–1000 cm^−1^. All Raman spectra were fitted using Gaussian multi-peak functions in Origin 2022 (OriginLab Corporation, Northampton, MA, USA) after baseline subtraction. The chemical states of Fe, Cr, and O on the specimen surfaces were analyzed by X-ray photoelectron spectroscopy (ESCALAB QXi, Thermo Fisher Scientific, Waltham, MA, USA). An Al Kα X-ray source was used for excitation, with a spot size of 500 μm and a pass energy of 150 eV for survey-scan acquisition. Each XPS spectrum was obtained by averaging three scans. Depth-dependent spectra were further collected by ion sputtering, and the sputtering rate was estimated from literature values [13].

### 2.4. Synchrotron Grazing-Incidence X-Ray Diffraction (GIXRD)

Synchrotron GIXRD measurements were performed at the BL15U (BL15U beamline, Shanghai Synchrotron Radiation Facility, Shanghai, China) beamline of the Shanghai Synchrotron Radiation Facility (SSRF). The photon energy of the incident X-ray beam was 20 keV, corresponding to a wavelength of λ = 0.6204 Å. The incident angle was increased from 0.05° to 0.55° in steps of 0.05°. The X-ray exposure time for each measurement was 5 s. Diffraction signals were collected using an MX225 (Rayonix, L.L.C., Evanston, IL, USA) area detector. The experimental data were converted using Dioptas 0.8.4 software (Clemens Prescher, University of Freiburg, Freiburg, Germany). For phase identification and comparison with standard reference patterns, the diffraction profiles were converted to Cu Kα-equivalent patterns (λ = 1.5406 Å) and further analyzed using Jade 9.0 software (Materials Data, Inc., Livermore, CA, USA).

### 2.5. Transmission Electron Microscopy (TEM)

To determine the thickness and composition of the passive film, transmission electron microscopy (TEM) specimens were prepared by focused ion beam milling using a Talos F200X G2 TEM/FIB system (Thermo Fisher Scientific, Waltham, MA, USA). For site-specific cross-sectional milling, a 30 kV Ga^+^ ion beam was first used for rapid material removal, followed by fine polishing at a reduced beam voltage of 2 kV. Secondary electron imaging in the FIB system was conducted using a 5 kV field-emission electron source. The prepared cross-sectional lamellae were mounted on OmniProbe^®^ copper grids (Thermo Fisher Scientific, Waltham, MA, USA) for subsequent observation. High-resolution transmission electron microscopy (HRTEM) was performed using an Talos F200X G2 transmission electron microscope (Thermo Fisher Scientific, Waltham, MA, USA) operated at an accelerating voltage of 200 kV. Elemental analysis was conducted using an energy-dispersive X-ray spectroscopy system (Bruker Scientific LLC, Billerica, MA, USA).

### 2.6. Electrochemical Measurements

Electrochemical experiments were conducted using an electrochemical workstation equipped with a three-electrode cell (Interface 1010E, Gamry Instruments, Warminster, PA, USA), with at least three replicate measurements performed for each condition. Electrochemical tests were carried out on both unexposed stainless-steel specimens and stainless-steel specimens exposed at the two Antarctic stations. The specimens served as the working electrode (exposed area: 1 cm^2^), while a platinum electrode and an Ag/AgCl electrode were used as the counter electrode and reference electrode, respectively. A 3.5 wt.% NaCl solution was employed as the electrolyte. Electrochemical impedance spectroscopy (EIS) measurements were performed over a frequency range from 10^5^ to 10^−2^ Hz with an AC perturbation amplitude of 5 mV. Mott–Schottky measurements were conducted at a frequency of 100 Hz, using a potential step of 50 mV and a potential scanning range of ±600 mV relative to the open-circuit potential (OCP).

## 3. Result and Discussion

### 3.1. The Environmental Characteristics of Antarctica

**Figure 1:**
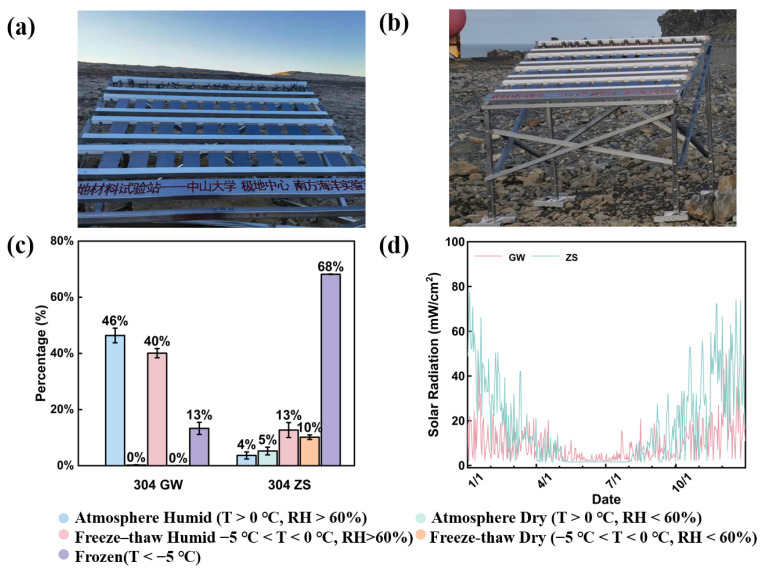
Field exposure facilities and environmental characteristics at the Antarctic test sites: (**a**) Great Wall Station and (**b**) Zhongshan Station; (**c**) average polar environmental spectra from 2021 to 2023 of Great Wall Station and Zhongshan Station; (**d**) average solar irradiance from 2021 to 2023.

Figure 1a,b show the atmospheric corrosion exposure facilities deployed at the Great Wall and Zhongshan Stations, respectively. The exposure racks were installed at an inclination angle of 45°. 304 stainless steel was used as the test material. The specimens were mounted using polytetrafluoroethylene rails, inserted into grooves, and uniformly separated and fixed by polytetrafluoroethylene spacers to form individual exposure units. At the same time, because polytetrafluoroethylene (PTFE) is an electrically insulating material, galvanic corrosion was effectively prevented. The specimen frames were fabricated from stainless steel, with each rack designed to accommodate multiple exposure units. After three years of field exposure, the devices were retrieved, sealed, packaged, and transported to the laboratory for subsequent characterization and analysis.

The environmental parameters were classified according to ISO 9223:1992 [14], and Figure 1c present the environmental spectra for Great Wall and Zhongshan Stations, respectively. The two sites exhibited markedly different environmental characteristics. Great Wall Station had an annual mean temperature of approximately −0.9 °C, relatively high humidity, and higher annual precipitation than Zhongshan Station. Frozen conditions mainly occurred during autumn and winter; more than 45% of the annual exposure time corresponded to an atmospheric state, while more than 35% was associated with freeze–thaw conditions. In contrast, Zhongshan Station was characterized by a cold and dry climate, with an annual mean temperature of approximately −12 °C. Owing to the influence of local topography, strong winds were frequent, with an annual mean wind speed of 6–8 m s^−1^ and more than 100 gale days per year. Frozen conditions accounted for more than 60% of the annual exposure time, whereas other surface states mainly occurred during the warmer summer and autumn periods and showed more frequent transitions within a single day.

Although low temperature and electrolyte isolation can partially suppress corrosion reactions, the Antarctic environment involves highly coupled and rapidly changing surface states. These coupled conditions may still damage the passive film and trigger localized corrosion. As shown in Figure 1d, high-solar-radiation periods were concentrated mainly in summer. Zhongshan Station also experiences polar day conditions, leading to higher solar irradiance during this period.

### 3.2. Pitting Corrosion Behaviors

**Figure 2:**
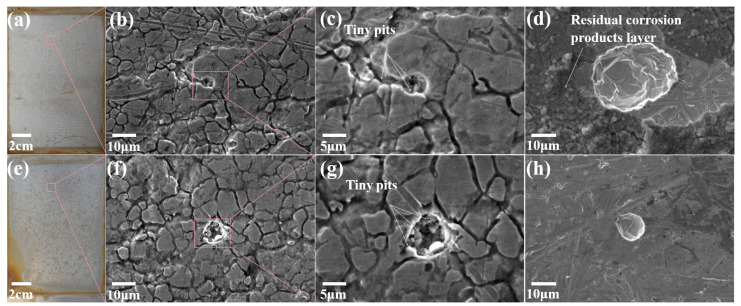
Corrosion morphologies of 304 stainless steel exposed to the Antarctic atmosphere. (**a**) Macroscopic appearance of 304 GW; (**b**,**c**) SEM morphologies of 304 GW before removal of corrosion products; (**d**) SEM morphology of 304 GW after acid pickling; (**e**) macroscopic appearance of 304 ZS; (**f**,**g**) SEM morphologies of 304 ZS before removal of corrosion products; and (**h**) SEM morphology of 304 ZS after acid pickling.

Figures S1 and S4 shows the surface morphology of the 304 Blank specimen, on which no obvious corrosion features were observed, except for a few machining scratches. In contrast, Figure 2a,e show the macroscopic corrosion appearances of the 304 stainless steel specimens after three years of exposure at Great Wall and Zhongshan Stations in Antarctica, respectively. After outdoor exposure, clear evidence of corrosion was observed on the stainless steel surfaces. The original metallic luster was almost completely lost, and the corrosion morphology was dominated by pitting. The pits were relatively uniformly distributed across the specimen surfaces. The corrosion products appeared as yellow-brown spots or flake-like deposits and were heterogeneously distributed, with a higher density generally observed near the specimen edges. This feature may be related to preferential melting of snow and ice from the specimen edges toward the center, resulting in longer surface-water-film retention at the edges and consequently more severe corrosion [15]. Numerous small pits were also observed on both 304 GW and 304 ZS. These pits were frequently associated with surface defects, particularly machining scratches, which can serve as pit-initiation sites.

Figure 2b–d,f–h present the surface morphologies of 304 stainless steel exposed at Great Wall and Zhongshan Stations before and after removal of corrosion products. The surfaces were mainly covered by blocky and lamellar corrosion products, accompanied by cracks and corrosion pits. As shown in Figure 2b,c,f,g, secondary micro-pits were present inside some pre-existing pits after exposure. In addition, the EDS results shown in Figures S2 and S3 reveal the presence of Cl-containing species inside the corrosion pits of both 304 GW and 304 ZS after three years of exposure, indicating that chloride ions were involved in the pitting process under the Antarctic atmospheric environment. The accumulation of corrosion products on the surface also induced cracking. After removal of the corrosion products, the pit walls of 304 GW appeared rougher, with more pronounced corrosion traces (Figure 2d). In contrast, the pit walls of 304 ZS were relatively smoother (Figure 2h). Interestingly, 304 ZS exhibited an anisotropic corrosion morphology. The SEM images in Figure 2d,h reveal multiple flat crystallographic facets on the corroded pit walls, suggesting preferential corrosion along specific crystallographic planes. Because the microscopic corrosion behavior of steels can be influenced by the crystallographic orientation and chemistry of MnS inclusions, sulfide species in Antarctic atmospheric deposits may have participated in pitting on 304 stainless steel [16,17].

**Figure 3:**
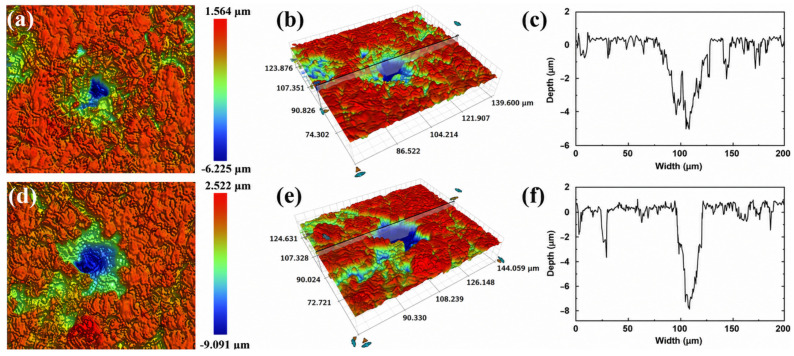
White-light interferometric images, three-dimensional morphologies, and cross-sectional profiles of corrosion pits on 304 stainless steel: (**a**–**c**) 304 GW and (**d**–**f**) 304 ZS.

As shown in Figure 3, the microscopic morphologies of 304 GW and 304 ZS further confirm that pitting was the dominant corrosion mode for 304 stainless steel at both Antarctic stations. Most pits formed under the Antarctic atmospheric environment were irregular and shallow, as shown in Figure 3a,d. The pits on 304 GW were mainly characterized by a wide and shallow morphology. Their cross-sectional profiles were conical, extending gradually from the upper edge toward the pit bottom, as shown in Figure 3b,e. The pit-wall inclination measured by optical profilometry was approximately 8–12°. In contrast, the pits on 304 ZS exhibited steeper pit walls, with inclination angles of approximately 13–18°, as determined from three-dimensional profile analysis, as shown in Figure 3c,f. Therefore, the pits on 304 ZS can be classified primarily as vertically developed pits.

The corrosion rates of the 304 stainless steel specimens exposed at Great Wall and Zhongshan Stations were calculated using the mass-loss method after standardized removal of corrosion products, and the pitting parameters are summarized in Table 2. The term “mass loss” in Table 2 refers to the net decrease in metallic substrate mass after removal of corrosion products, soluble salts, and loose deposits, rather than to the gross mass of the rust-covered specimen immediately after exposure. Therefore, the uptake of oxygen and water into oxide/hydroxide corrosion products is not inconsistent with the measured mass decrease, because these corrosion products were intentionally removed before the final weighing. During atmospheric exposure, thin electrolyte films generated by deposited sea salts, snow/ice melting, and freeze–thaw cycling can promote localized anodic dissolution of alloying elements and the formation of corrosion products [18,19]. A portion of the soluble corrosion species and chloride-containing salts can dissolve into meltwater or brine films and be transported away as the inclined specimens drain. In addition, weakly adherent or porous corrosion products may be naturally removed or redistributed by meltwater, ice movement, wind, and wet–dry cycling during unsheltered exposure [20,21]. Such loss or redistribution of soluble and loose corrosion products is a common feature of atmospheric corrosion under outdoor exposure conditions. After retrieval, the remaining adherent corrosion products were removed by standardized chemical cleaning to determine the net loss of metallic substrate. The average corrosion rates were approximately 1.428 μm year^−1^ for 304 GW and 1.643 μm year^−1^ for 304 ZS. Under Antarctic atmospheric exposure, both uniform corrosion and pitting contributed to the mass loss of the specimens. However, pitting is more detrimental than uniform corrosion because it can accelerate localized failure [16]. The average pit depths of 304 GW and 304 ZS were of the same order of magnitude, with the Zhongshan specimens showing slightly deeper pits in the range of 4–6 μm. Nevertheless, the maximum pit depth of 304 ZS (8.24 μm) was much greater than that of 304 GW (5.51 μm). The pitting factors of 304 GW and 304 ZS, defined as the ratio of maximum pit depth to the average corrosion penetration calculated from the three-year mass loss, were approximately 1.29 and 1.67, respectively. The average pit widths of 304 GW and 304 ZS were 27.09 ± 8.73 μm and 31.69 ± 11.32 μm, respectively, and both the average and maximum pit widths of 304 ZS were larger than those of 304 GW. One-way ANOVA using exposure site as the fixed factor confirmed that pit depth differed significantly between the two sites (F(1, 30) = 17.05, *p* = 2.67 × 10^−4^), whereas the pit-width difference was not statistically significant at the 95% confidence level (F(1, 30) = 0.60, *p* = 0.447). Under concentrated liquid-film conditions beneath ice, particularly when chloride ions are enriched around pits, lateral pit growth can exceed growth in the depth direction [22,23]. The pit densities of 304 GW and 304 ZS were 32.47 cm^−2^ and 43.31 cm^−2^, respectively. The higher pit density of 304 ZS indicates a higher pit-initiation frequency at Zhongshan Station, leading to more rapid localized corrosion. In the complex Antarctic atmospheric environment, sea-salt aerosol deposition and frequent freeze–thaw cycling at Zhongshan Station can promote chloride enrichment on the metal surface and facilitate the formation of thin electrolyte films, thereby accelerating pit initiation and propagation [24,25].

### 3.3. Composition of Corrosion Products (Raman Analysis)

**Figure 4:**
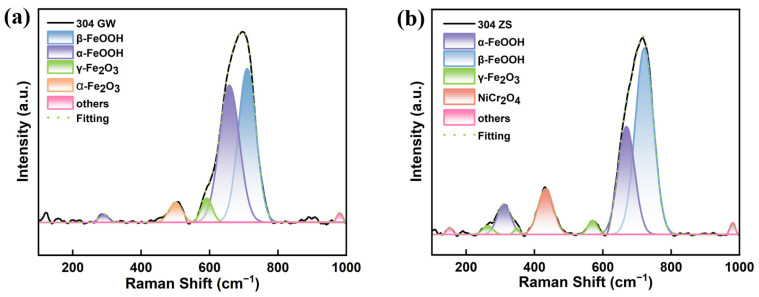
Raman spectra acquired from corrosion products in pits on 304 stainless steel: (**a**) 304 GW and (**b**) 304 ZS.

To identify the corrosion-product composition, Raman spectroscopic analysis was performed on residual corrosion products retained inside pits on the 304 stainless steel specimens. As shown in Figure 4, the products formed during pitting were mainly β-FeOOH, α-FeOOH and γ-Fe_2_O_3_. Previous studies have shown that corrosion products formed under atmospheric exposure are generally dominated by ferric phases, whereas ferrous phases are less stable [26]. Consistent with the present results, no clear Raman signal corresponding to divalent iron species was detected, suggesting that the corrosion products formed under Antarctic atmospheric conditions were mainly ferric oxides or oxyhydroxides. The dominant products in the pits of 304 GW were α-FeOOH and β-FeOOH (Figure 4a), whereas β-FeOOH was the principal product in the pits of 304 ZS (Figure 4b). In chloride-rich aqueous environments, Fe(III) species can be induced by Cl^−^ to form β-FeOOH. When the Cl^−^ concentration is below a critical threshold, Fe(III) species tend to form α-FeOOH, the thermodynamically most stable naturally occurring iron oxyhydroxide [27]. At Zhongshan Station, freeze–thaw cycling may produce chloride-concentrated solution beneath ice, enhancing Cl^−^ mobility and reactivity and thereby favoring β-FeOOH formation. By contrast, at Great Wall Station, the Cl^−^ concentration in the humid surface electrolyte film may have been lower or less continuously replenished, resulting in a higher relative proportion of α-FeOOH. It should be noted that, although EDS results revealed the presence of Cl-containing species inside the corrosion pits, the present Raman, XPS, GIXRD, and TEM-EDS analyses did not provide reliable quantitative confirmation of residual chloride or chlorine-containing species on the cleaned specimens. Therefore, the role of chloride in the proposed corrosion mechanism is treated as an inference based on the Antarctic marine-atmospheric exposure environment, the literature on chloride-assisted pitting of 304 stainless steel, and the preferential formation of β-FeOOH, rather than as a direct chemical quantification of chloride in the present specimens. At Zhongshan Station, freeze–thaw cycling may produce chloride-concentrated solution beneath ice, enhancing Cl^−^ mobility and reactivity and thereby favoring β-FeOOH formation. By contrast, at Great Wall Station, the Cl^−^ concentration in the humid surface electrolyte film may have been lower or less continuously replenished, resulting in a higher relative proportion of α-FeOOH.

### 3.4. Composition and Structural Analysis of the Passive Film (XPS, GIXRD and FIB-TEM)

**Figure 5:**
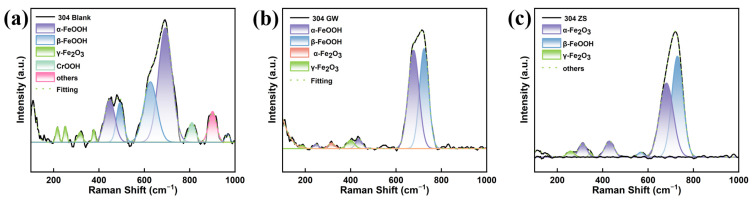
Raman spectra of the passive films formed on 304 stainless steel surfaces: (**a**) 304 Blank, (**b**) 304 GW, and (**c**) 304 ZS.

Raman spectroscopy and XPS were employed to analyze the chemical composition and elemental valence states of the surface oxide/passive layers formed on 304 Blank, 304 GW and 304 ZS. Figure 5 presents the Raman spectra of the three specimens. To minimize the influence of material-batch variation and initial surface condition, 304 Blank was used as the reference specimen. Its passive film was considered representative of the initial state of 304 GW and 304 ZS, thereby enabling Antarctic-exposure-induced changes to be identified. The main Raman peak positions of the three specimens were similar, indicating broadly comparable surface oxide species. Raman bands at approximately 200–300 cm^−1^, 400 cm^−1^ and 600–800 cm^−1^ suggest the presence of γ-Fe_2_O_3_, α-FeOOH and β-FeOOH on the 304 stainless steel surfaces [28]. Because Raman spectroscopy has limited sensitivity and depth resolution for ultrathin passive films, these spectra should be interpreted as reflecting the near-surface oxide/passive layer rather than the passive film alone. The slight difference between the Raman signatures in Figure 5 and the pit corrosion products in Figure 4 is therefore reasonable.

**Figure 6:**
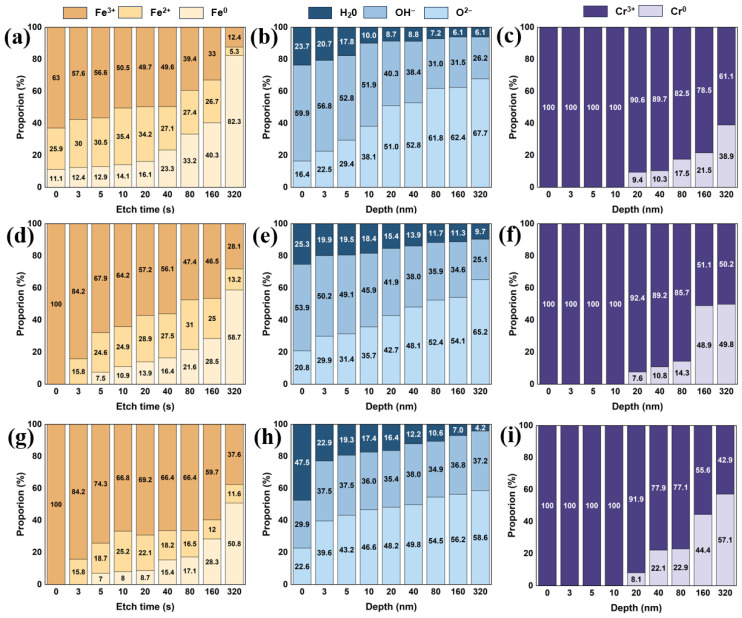
Valence-state analysis of Fe, O, and Cr by depth-resolved XPS for (**a**–**c**) 304 Blank, (**d**–**f**) 304 GW, and (**g**–**i**) 304 ZS.

The XPS results for the passive films formed on the stainless steel surfaces are shown in Figure 6. Detailed peak fitting and analysis were performed on the high-resolution Fe 2p, O 1s, and Cr 2p spectra. The surfaces of 304 GW (Figure 6d) and 304 ZS (Figure 6g) were dominated by ferric iron species, whereas 304 Blank (Figure 6a) exhibited ferric, ferrous, and metallic iron signals. With increasing sputtering depth, the relative content of ferric species gradually decreased in all three specimens. This trend reflects the oxide/hydroxide nature of the outer passive film and the progressive transition toward the metal-rich inner layer or substrate interface, where oxygen diffusion is restricted and the local chemical environment is less favorable for stable Fe^3+^ species [26].

The O 1s spectra can generally be deconvoluted into lattice oxygen, hydroxyl oxygen, and adsorbed or bound water components. The lower-binding-energy component is mainly associated with O^2−^ in metal oxides, such as lattice oxygen in Fe_2_O_3_ and Cr_2_O_3_. In contrast, the higher-binding-energy components are assigned mainly to OH^−^ and H_2_O, reflecting metal hydroxides and surface-adsorbed or structurally bound water, respectively. Stainless-steel passive films often exhibit a layered structure in which a hydrated, hydroxyl-enriched outer layer covers a more oxide-rich inner layer. Therefore, with increasing sputtering depth, the O 1s spectra show a transition from adsorbed/hydroxylated species (H_2_O/OH^−^) to lattice oxide species (O^2−^), indicating that the inner passive layer is more compact and more oxide-like, whereas the outer layer is relatively hydrated and enriched in environmental adsorbates [29].

For Cr-containing species on the steel surface, the spectra evolved from surface Cr^3+^ enrichment toward a Cr^0^-dominated state with increasing sputtering depth, reflecting progressive removal of the surface oxide film and approach to the uncorroded metallic substrate. Qualitative analysis of the high-resolution elemental spectra indicates that the passive films were composed mainly of FeOOH, Fe_2_O_3_, Cr_2_O_3_, and related species. These thermodynamically stable metal oxides and oxyhydroxides constitute the principal protective components of the passive film and provide corrosion resistance to the stainless steel substrate.

**Figure 7:**
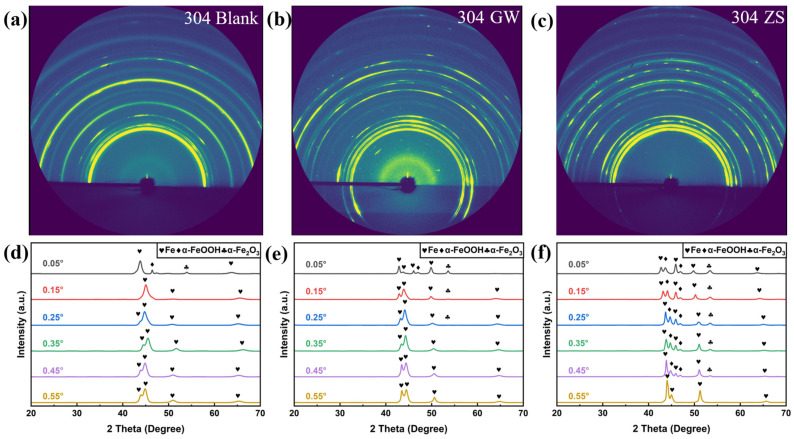
(**a**–**c**) GIXRD patterns recorded at a grazing-incidence angle of 0.05°; (**d**–**f**) integrated GIXRD patterns at all incidence angles.

The crystal structure of the passive films on 304 Blank, 304 GW, and 304 ZS was characterized by GIXRD. At the lowest incidence angle of 0.05°, the diffraction signal was highly surface-sensitive; the nominal penetration depth was estimated to be approximately 0.2 nm and should be verified against the absorption and geometry used in the experiment. The diffraction patterns of 304 Blank, 304 GW, and 304 ZS are shown in Figure 7. The patterns contained both distinct polycrystalline diffraction rings and diffuse amorphous scattering, indicating that the passive film on 304 stainless steel was not a single crystalline structure but consisted of crystalline/nanocrystalline phases together with an amorphous phase. This result is consistent with the accepted bilayer structure of stainless-steel passive films and the presence of Fe/Cr oxide-hydroxide composite phases [30,31,32]. Notably, amorphous scattering was more pronounced for 304 ZS, indicating that its passive film contained a larger amorphous fraction.

Figure 7d–f present the integrated GIXRD patterns of the passive films on 304 Blank, 304 GW, and 304 ZS. A broad hump in the low-angle region of all three spectra corresponds to the inner-ring amorphous scattering observed in the two-dimensional diffraction patterns. The diffraction peaks are mainly assigned to Fe_2_O_3_ (PDF #97-008-1248), FeOOH (PDF #97-003-3615), and Fe (PDF #97-063-1722), in agreement with the Raman results. Differences in peak intensity suggest differences in phase abundance, film thickness, or preferred orientation rather than composition alone. 304 Blank and 304 GW showed similar peak shapes and positions, whereas 304 ZS exhibited a stronger FeOOH contribution. The principal diffraction peaks were slightly shifted relative to the standard PDF reference patterns, which may be associated with residual stress or lattice distortion in the thin film or near-surface layer [33]. When the grazing-incidence angle was increased to 0.55°, the corresponding nominal penetration depth was approximately 0.388 μm, extending into the substrate region. Under these conditions, the spectra of 304 Blank, 304 GW, and 304 ZS nearly overlapped, and the diffraction peaks were mainly attributed to Fe (PDF #97-017-1002). Thus, Antarctic exposure had limited influence on the crystal structure of the 304 stainless steel substrate; its effects were concentrated mainly in the outermost nanometer- to submicrometer-scale passive film and corrosion-product layer.

**Figure 8:**
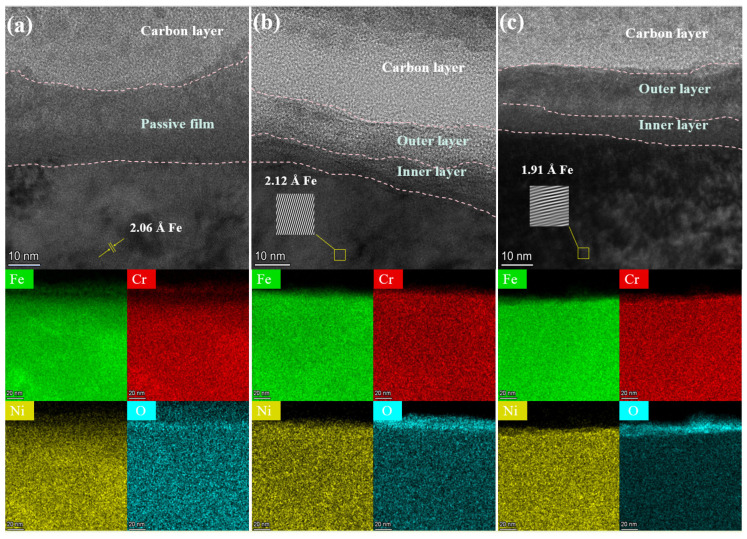
Cross-sectional HRTEM and EDS images of (**a**) 304 Blank, (**b**) 304 GW, and (**c**) 304 ZS.

To further reveal passive-film microstructural evolution after Antarctic atmospheric exposure, FIB cross-section preparation followed by HRTEM/EDS analysis was performed on 304 Blank, 304 GW, and 304 ZS. Before FIB milling, an amorphous carbon protective layer was deposited on the specimen surface to minimize ion-beam damage to the ultrathin passive film. Owing to the contrast among the carbon layer, passive film, and 304 stainless steel substrate, these regions could be clearly distinguished in cross-section. Figure 8a shows that the passive film on 304 Blank was complete, continuous, and uniform, with a total thickness of approximately 27 nm, indicating that the unexposed specimen retained a relatively stable native air-formed passive state. In contrast, the passive films on the exposed specimens underwent structural reconstruction. The film on 304 GW exhibited a clear bilayer structure consisting of outer and inner layers (Figure 8b), whereas the layered feature in 304 ZS was less sharply resolved (Figure 8c).

For 304 GW, the outer passive-film layer was approximately 6–8 nm thick, and the inner layer was approximately 8–10 nm thick (Figure 8b). The two layers showed clear contrast differences: the outer layer was relatively porous and had lower contrast, whereas the inner layer was denser and exhibited higher contrast. This bilayer structure is closely associated with selective dissolution and redeposition under surface thin-film conditions [33]. In the atmosphere, when the passive film on 304 stainless steel is damaged, film growth toward the substrate occurs at the passive-film/substrate interface while the film is thinned. In chloride-rich solutions, Fe ions can precipitate as β-FeOOH. Under wet–dry cycling and chloride-containing thin liquid films, Fe dissolves preferentially and Cr becomes relatively enriched in the inner layer. As exposure proceeds, hydroxylated products accumulate in the outer layer, forming a bilayer structure distinct from the air-formed film. This mechanism is consistent with the cross-sectional contrast observed for 304 GW, namely a porous outer layer and a dense inner layer [34].

The passive film on 304 ZS had a relatively uniform thickness of approximately 20 nm, with an outer layer of about 11–12 nm and an inner layer of about 6–9 nm (Figure 8c). After three years of Antarctic atmospheric exposure, the passive-film thickness on 304 ZS decreased by approximately 26% relative to 304 Blank. The contrast of the passive film on 304 ZS was similar to that on 304 Blank, indicating that its outer-layer microstructure was less porous than that of 304 GW. Moreover, the interface between the outer and inner layers was not sharply resolved. The outer layer resembled the native passive film formed on 304 stainless steel in air, suggesting that it may represent residual air-formed passive film, whereas the inner layer may be a newly formed film generated during passive-film growth.

The differences in passive-film thickness among the three specimens are also consistent with the XPS observations. Compared with 304 GW, the thicker passive film on 304 ZS more effectively attenuated the XPS signal from the 304 stainless steel substrate. As reflected by the Fe 2p spectra (Figure 6a,d,g), the metallic Fe contribution was strongest for 304 Blank, followed by 304 GW and then 304 ZS. These results indicate that Antarctic exposure modified the oxide/metal signal ratio by changing passive-film thickness and structure. The Fe^3+^ fraction in the oxide layer was higher than the Fe^2+^ fraction, suggesting that Antarctic atmospheric exposure promoted Fe oxidation and the formation of stable ferric oxides.

TEM-EDS was used to analyze the elemental compositions of the substrate and passive film of the 304 stainless steel specimens; the results are summarized in Table 3. The atomic ratios of the principal alloying elements Fe, Cr, and Ni in the substrates of 304 Blank, 304 GW, and 304 ZS were generally similar, indicating that exposure at Great Wall and Zhongshan Stations did not measurably alter the bulk alloy composition. Because the native passive film on 304 stainless steel is extremely thin and the interfaces between layers are not sharply defined, it was difficult to accurately select separate positions for the inner and outer layers. Therefore, layer-by-layer elemental compositions could not be reliably quantified at this stage.

Regarding passive-film composition, the Fe content of the film on 304 GW was clearly higher than that on 304 Blank, whereas 304 ZS showed only a slight increase. The O atomic fraction was substantially higher in the passive film than in the substrate, consistent with the formation of oxide/hydroxide layers through reactions between surface metals and oxygen or water in the environment [35]. Under chloride-containing wet–dry and freeze–thaw conditions, the Fe-enriched outer layer is inferred to be more susceptible to selective dissolution or migration during environmental attack [36]. Because Cr preferentially participates in passivation and forms stable Cr_2_O_3_, local Cr enrichment can occur in the passive film. For 304 GW, the Cr content increased from 17.69 at.% in the substrate to 21.96 at.% in the passive film. The lower measured Cr fraction in the passive films of 304 Blank and 304 ZS may result from local sampling of ultrathin films, thickness fluctuations, and volume-averaging effects. At Great Wall Station, freeze–thaw cycling likely promoted local damage and repassivation of the outer film, shifting the film/substrate interface toward the substrate and forming a new Cr-rich inner layer. Meanwhile, part of the Fe-rich oxide/hydroxide outer layer may have dissolved, migrated, or spalled, leading to a higher overall Cr fraction and a thinner total film. Ni has a lower tendency to oxidize than Fe and Cr and therefore remains mainly in the metallic substrate during passivation, with only a small amount incorporated into the passive film. Consequently, the Ni atomic ratio changed only slightly between the passive film and substrate and remained approximately 6–7.5% [37].

### 3.5. Electrochemical Properties of 304 Stainless Steel

Atmospheric corrosion of metals is fundamentally an electrochemical process occurring beneath surface liquid films. Therefore, EIS and Mott–Schottky methods were used to evaluate changes in the electrochemical and semiconducting properties of the passive film on 304 stainless steel after three years of exposure at Great Wall and Zhongshan Stations.

**Figure 9:**
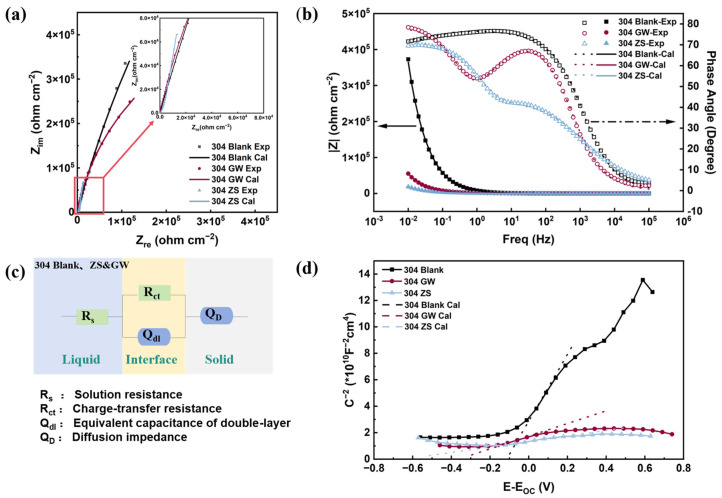
Electrochemical spectra of 304 Blank, 304 GW, and 304 ZS: (**a**) Nyquist plots, (**b**) Bode plots, (**c**) equivalent-circuit model used for EIS fitting, and (**d**) Mott–Schottky plots.

Figure 9 shows the EIS and Mott–Schottky results for the 304 stainless steel specimens. To evaluate the electrochemical behavior of specimens with different surface states, the EIS data were fitted using an equivalent-circuit model containing two relaxation processes (Figure 9c). The fitted parameters include solution resistance (R_s_), the double-layer constant phase element (Q_dl_), the double-layer dispersion exponent (n_1_), charge-transfer resistance (R_ct)_, the diffusion-related constant phase element (Q_d_), and the diffusion dispersion exponent (n_2_). In Figure 9a, all Nyquist plots show capacitive behavior, and distinct capacitive arcs are observed for 304 GW and 304 ZS. Figure 9b shows the Bode plots, in which the horizontal axis is frequency and the left and right vertical axes represent impedance magnitude (solid lines) and phase angle (dashed lines), respectively. Low-frequency impedance generally reflects the polarization resistance of the electrode/passive-film system, whereas high-frequency impedance is dominated by solution resistance. After three years of Antarctic exposure, the radii of the capacitive arcs decreased, indicating reduced charge-transfer resistance and lower passive-film stability. The |Z| value at 0.01 Hz also decreased for both exposed specimens, confirming reduced corrosion resistance. In addition, a larger and broader phase-angle region at medium frequencies corresponds to a more stable passive film; after exposure, this region became smaller and narrower, indicating degradation of passive-film quality. The fitted results are listed in Table 4. R_ct_ decreased by orders of magnitude after Antarctic exposure, indicating that charge transfer became easier and that the protective resistance of the passive film decreased markedly [38].

The passive film on 304 stainless steel consists primarily of Fe and Cr oxides and hydroxides; therefore, its semiconducting properties strongly influence film conductivity and the dominant point-defect. Figure 9d shows the Mott–Schottky curves of the specimens. The horizontal axis is the potential relative to the open-circuit potential, denoted E − E_OCP, and the vertical axis is the inverse square of the film capacitance, C^−2^. Within ±0.6 V of the open-circuit potential, the Mott–Schottky curves exhibit multiple linear regions, indicating a heterogeneous passive-film structure in which different potential ranges correspond to different film regions. FIB-TEM and XPS results support a bilayer or compositionally graded passive film; thus, the multiple linear regions in the Mott–Schottky curves reflect heterogeneity in the electronic structure of the film [33]. In the positive-slope region near the open-circuit potential, the passive film behaves predominantly as an n-type semiconductor, with electrons as the main carriers. Accordingly, donor density was used to describe the dominant defect population. From the point-defect-model perspective, this behavior indicates donor-type defects such as oxygen vacancies or metal-cation interstitials [34]. The donor density (N_D_) and flat-band potential (E_fb_) of the passive film were calculated using the Mott–Schottky equation [39,40,41,42]:

(1) $$\frac{1}{C^{2}}=\frac{2}{\varepsilon \varepsilon_{0}eN_{D}}\left(E-E_{fb}-\frac{kT}{e}\right)$$

where C is the capacitance of the passive film, E is the applied potential relative to Eocp, ε_0_ is the vacuum permittivity (8.854 × 10^−14^ F cm^−1^), ε is the relative permittivity of the passive film, taken as 15.6 [27], e is the elementary charge (1.602 × 10^−19^ C), k is the Boltzmann constant (1.38 × 10^−23^ J K^−1^), and T is the thermodynamic temperature (K).

According to Equation (1), N_D_ and E_fb_ were calculated from the slope and intercept of the linear regions in the Mott–Schottky plots; the results are summarized in Table 5. The N_D_ of the passive film on 304 Blank was lower than that on 304 GW, whereas N_D_ for 304 ZS was slightly higher than that for 304 GW. These results indicate that Antarctic atmospheric exposure, especially at Zhongshan Station, substantially increased the concentration of donor-type defects in the passive film. Because carrier density largely controls semiconductor conductivity, 304 ZS is expected to exhibit the lowest R_ct_ in EIS measurements, followed by 304 GW and 304 Blank. According to the point-defect model, passive-film growth and dissolution are controlled by the generation, migration, and annihilation of point defects at the film/solution and film/metal interfaces. A steady-state passive film generally consists of a dense barrier layer near the metal and an outer deposited layer. In chloride-containing environments, Cl^−^ can alter film chemistry and defect structure, making the passive state less stable, less resistive, and more conductive [43,44].

In the Antarctic atmosphere, passive-film dissolution is unavoidable and is accompanied by oxygen-vacancy generation. For 304 GW, mild Cl^−^ attack at Great Wall Station likely caused partial substitution of oxygen sites by Cl^−^ in the film, inducing a limited number of oxygen vacancies and increasing the donor-defect density to 3.35 × 10^21^ cm^−3^. For 304 ZS, continuous Cl^−^ penetration coupled with UV-induced lattice degradation at Zhongshan Station likely led to more extensive oxygen-vacancy accumulation. In addition, wind erosion may have promoted repeated formation of highly defective oxide films, ultimately increasing N_D_ to 5.97 × 10^21^ cm^−3^.

The Mott–Schottky results further clarify the role of the flat-band potential (E_fb_). The passive film can be classified primarily as an n-type semiconductor. When an n-type semiconductor contacts an electrolyte, charge redistribution occurs to establish interfacial energy-level equilibrium. Under typical depletion conditions, a positively charged space-charge layer forms near the film surface and causes upward band bending. E_fb_ describes this interfacial electronic structure. A more negative E_fb_ is generally associated with stronger band bending and can favor electrostatic interaction with anions such as Cl^−^ [45]. The measured E_fb_ values followed the order 304 Blank > 304 GW > 304 ZS, indicating that the interfacial electronic structure of the passive film changed after exposure at both stations, with the most pronounced change at Zhongshan Station. Together with the increase in N_D_, this result suggests that the passive film on 304 ZS contained a higher defect concentration and was more susceptible to Cl^−^ induced local destabilization [34].

### 3.6. Mechanism of Low-Temperature Atmospheric Corrosion in Antarctica

**Figure 10:**
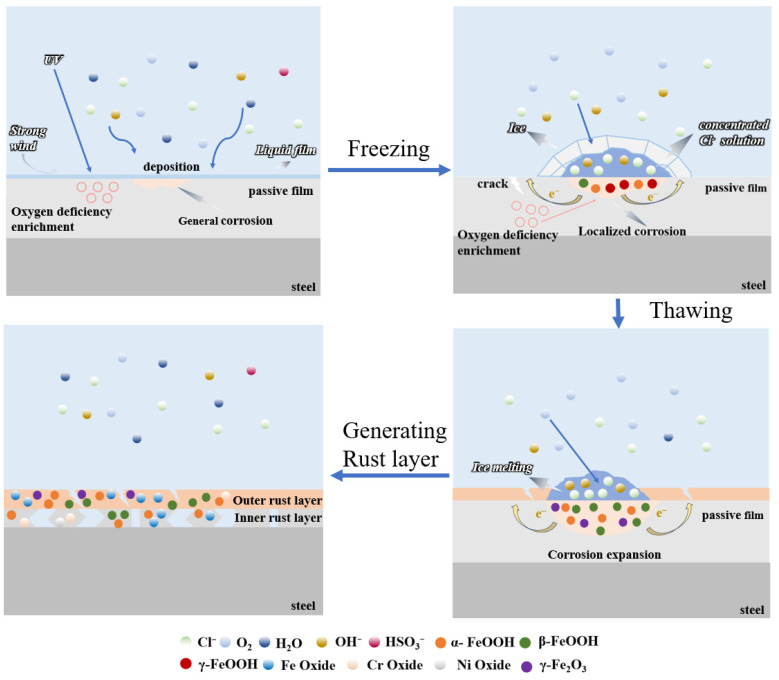
Schematic illustration of the proposed corrosion mechanism.

The atmospheric corrosion mechanism of 304 stainless steel showed a distinct site-dependent gradient between Great Wall and Zhongshan Stations. This difference originates from the coupled effects of temperature, sea-salt aerosol deposition, surface electrolyte-film state, and freeze–thaw processes at the two sites. Meteorological and environmental observations indicate that the northern Antarctic Peninsula region, where Great Wall Station is located, is generally milder than the East Antarctic coastal region around Zhongshan Station, whereas Zhongshan Station is colder. Sea-salt aerosols at both stations provide a plausible environmental source for chloride deposition and subsequent liquid-film formation on specimen surfaces [46]. Because residual chloride was not directly quantified on the cleaned specimens, the chloride-related steps in the following mechanism should be regarded as a proposed pathway supported by environmental conditions, corrosion morphology, and literature evidence, rather than as direct proof of measured chloride-containing phases in this work. The proposed mechanism is shown schematically in Figure 10.

After marine aerosols are deposited on 304 stainless steel by wet and dry deposition, the relatively higher temperature, sea fog, and sea-salt deposition at Great Wall Station are more likely to maintain a thicker liquid electrolyte film for extended periods. Corrosion there therefore resembles conventional atmospheric corrosion beneath a thin electrolyte film. In contrast, under the lower-temperature conditions at Zhongshan Station, localized surface electrolyte films can persist beneath snow or ice, and freezing-induced concentration more readily enriches Cl^−^ in localized regions. Studies of 304 stainless steel beneath thin chloride-containing electrolyte layers have shown that decreasing electrolyte-layer thickness shifts the pitting potential negatively, decreases charge-transfer resistance, and increases the number of small pits; under lower relative humidity or higher salt concentration, atmospheric pitting is more easily triggered and localized corrosion becomes dominant [47,48]. The metal surface inside a pit is activated and has a lower electrode potential, whereas the passive film surrounding the pit has a higher electrode potential. A micro-galvanic cell is therefore established between the pit interior and exterior (Figure 10). The anodic reaction (Equation (2)) occurs inside the pit, where Fe releases two electrons to form Fe^2+^. The cathodic reaction occurs outside the pit. Because dissolved oxygen is present in the thin electrolyte film and the Antarctic atmosphere, oxygen reduction is the dominant cathodic process (Equation (3)). Oxygen diffusion controls the cathodic reaction rate and therefore influences pit propagation. The slower pit propagation at Great Wall Station may be associated with greater mass-transfer resistance through a relatively thicker electrolyte film.

Anode: Fe (s) → Fe^2+^ (aq) + 2e^−^ (2)

Cathode: O_2_ (g) + 2H_2_O (l) + 4e^−^ → 4OH^−^ (aq) (3)

As temperature decreases and the surface enters the snow- and ice-covered stage, the snow/ice layer at Great Wall Station is relatively thin. During freezing, water solidifies only moderately preferentially, producing a weakly concentrated electrolyte environment and leaving more residual liquid electrolyte. Corrosion therefore remains closer to uniform corrosion; oxygen diffuses at a moderate rate through the thin snow/ice layer, ion channels formed by ice crystals are limited, and pit initiation is not pronounced. At Zhongshan Station, the lower temperature produces a thicker and denser snow/ice layer. During freezing, preferential water solidification is stronger, and the residual electrolyte becomes highly concentrated, enabling under-ice corrosion to continue at lower temperatures. Strong UV radiation associated with ozone depletion can damage the passive-film lattice, while frequent severe wind erosion can remove surface corrosion products and repeatedly expose fresh substrate. Oxygen can reach the substrate through porous snow and ice, and impurities together with ice crystals form ion-transport channels that accelerate the migration of Cl^−^ and OH^−^ toward the substrate interface. Together with strong Cl^−^ enrichment during freezing, Fe^2+^ can transform through hydrolyzed intermediates such as FeOH^+^ and preferentially form γ-FeOOH [49], as schematically represented in Equation (4). Some intermediates may further transform into more stable Fe_2_O_3_ through hydroxide formation and dehydration, as schematically represented in Equation (5).

Fe^2+^ (aq) → FeOH^+^ (aq) → γ-FeOOH (s) (4)

Fe^2+^ (aq) → Fe(OH)_2_ (s) → Fe(OH)_3_ (s) → Fe_2_O_3_ (s) (5)

When temperature increases, the ice layer begins to melt and a rust layer develops; pits then enter a propagation stage and continue to nucleate and grow. γ-FeOOH can transform through chloride-containing ferric oxyhydroxide intermediates, FeO_x_(OH)_3−2x_Cl, and ultimately form stable α-FeOOH, as schematically expressed in Equation (6). The diffusion of dissolved oxygen controls the cathodic reaction rate and hence the pit-propagation rate of 304 stainless steel. Therefore, owing to the greater mass-transfer resistance at Great Wall Station, pitting develops more slowly there than at Zhongshan Station.

γ-FeOOH (s) → FeO_x_(OH)_3−2x_Cl (s) → α-FeOOH (s) + HCl (aq) (6)

Salt-containing particles deposited from the Antarctic atmosphere continuously supply Cl^−^ to the thin liquid film on the material surface. Enrichment of Cl^−^ at the rust/metal interface lowers the freezing point and promotes local acidification, leading to formation of β-Fe_2_(OH)_3_Cl. This phase can subsequently transform through unstable green rust I (GRI) and finally yield β-FeOOH [50], as schematically shown in Equations (7) and (8). Under acidic interfacial conditions, hydrogen evolution can contribute to the cathodic reaction (Equation (9)), further accelerating pitting at the metal/rust interface.

2Fe^2+^ (aq) + 3OH^−^ (aq) + Cl^−^ (aq) → β-Fe_2_(OH)_3_Cl (s) (7)

β-Fe_2_(OH)_3_Cl (s) → GRI (s) → β-FeOOH (s) (8)

2H^+^ (aq) + 2e^−^ → H_2_ (g) (9)

As the rust layer thickens, differences in rust-layer stability and damage mechanisms between the two stations become more pronounced. At Great Wall Station, the amplitude of freeze–thaw-induced temperature fluctuation is moderate. Although the outer rust layer can adsorb water vapor, internal stress accumulation is limited, generating only a small number of cracks, and the relatively loose rust layer provides weak resistance to Cl^−^ penetration. At Zhongshan Station, strong UV degradation and wind erosion make the rust layer porous and loose. In addition, large temperature fluctuations make the supercooled substrate more prone to water-vapor adsorption. The water-absorption and storage capacity of the loose rust layer prolongs liquid-film residence. During freezing, volume expansion of solution trapped in the rust layer generates substantial internal stress and causes rust cracking; during thawing, melting ice crystals leave numerous cavities. Furthermore, differences in thermal expansion coefficient between the rust layer and substrate generate interfacial stresses during intense freeze–thaw cycling, ultimately leading to cracking or spallation at the rust/substrate interface.

Freeze–thaw cycling at both stations affects corrosion by regulating the surface liquid-film state, oxygen diffusion rate, and Cl^−^ concentration; however, the intensity of this effect differs between sites. At Great Wall Station, corrosion is influenced primarily by conventional freeze–thaw cycling, and the acceleration effect is moderate. At Zhongshan Station, freeze–thaw cycling acts as a distinctive form of low-temperature wet–dry cycling. Formation and melting of ice layers cause large fluctuations in liquid-film state, oxygen diffusion, and Cl^−^ concentration. During ice melting, oxygen diffusion increases markedly and a stable liquid film intensifies electrochemical reactions; during freezing, reduction of the liquid phase sharply increases Cl^−^ concentration, further accelerating pit growth. Consequently, freeze–thaw cycling promotes corrosion more strongly at Zhongshan Station than at Great Wall Station.

## 4. Conclusions

Based on three-year Antarctic field-exposure tests, environmental parameter statistics, passive-film structural characterization, and electrochemical measurements, this study investigated the corrosion behavior and passive-film evolution of 304 stainless steel at Great Wall and Zhongshan Stations. A mechanism for atmospheric corrosion of 304 stainless steel in Antarctica is proposed, and the coupling between passive-film destabilization and localized corrosion development is summarized as follows:

(1) 304 stainless steel underwent localized corrosion dominated by pitting at both Great Wall and Zhongshan Stations. The specimens exposed at Zhongshan Station exhibited higher mean and maximum pit depths and a higher pit density, indicating more severe overall corrosion than those exposed at Great Wall Station. The mean corrosion rates at Great Wall and Zhongshan Stations were 1.428 and 1.643 μm y^−1^, respectively. The mean/maximum pit depths were 4.16/5.51 μm at Great Wall Station and 5.85/8.24 μm at Zhongshan Station. The pit density at Zhongshan Station (43.31 cm^−2^) was also higher than that at Great Wall Station (32.47 cm^−2^).
(2) The corrosion products consisted mainly of β-FeOOH, α-FeOOH, and γ-Fe_2_O_3_. The β-FeOOH signature was more pronounced at the pit bottoms of the Zhongshan specimens, which is consistent with a stronger chloride-assisted corrosion tendency at this site.
(3) The Antarctic environment significantly altered the thickness and structure of the passive film on 304 stainless steel. The unexposed specimen (304 Blank) had a passive-film thickness of approximately 27 nm. After exposure, the film on 304 GW developed a clear bilayer structure, while the layer boundary in 304 ZS was less distinct. The film at Great Wall Station was approximately 14–18 nm thick, with an outer layer of 6–8 nm and an inner layer of 8–10 nm, whereas the film at Zhongshan Station was approximately 20 nm thick, with an outer layer of 11–12 nm and an inner layer of 6–9 nm. The passive films on all three specimens consisted of coexisting amorphous and nanocrystalline phases, but the Zhongshan specimen showed stronger amorphous scattering, indicating a higher degree of structural disorder.
(4) Antarctic exposure changed the structure and electrochemical properties of the passive film, reducing its electrochemical stability and increasing its defect concentration. Compared with 304 Blank, the exposed specimens exhibited substantially lower charge-transfer resistance, indicating diminished corrosion resistance and poorer passive-film stability, consistent with the field-exposure observations. The pronounced daily fluctuations in the polar environment promote repeated breakdown and regeneration of the passive film.
(5) The three-year field exposure results indicate that pitting of 304 stainless steel in Antarctica is governed by a thin-liquid-film-assisted mechanism under freeze–thaw atmospheric conditions. Based on the marine-atmospheric exposure conditions and the literature on chloride-assisted pitting, chloride deposition and intermittent snow/ice melting are inferred to promote the repeated formation of transient electrolyte films, which destabilize the passive film and provide localized electrochemical conditions for pit initiation and propagation. This mechanism links the observed pitting morphology, corrosion-product accumulation and passive-film degradation to the actual polar exposure environment.

## Figures and Tables

**Table 1:** Chemical composition of 304 SS determined before exposure by XRF (wt%).

Element	Mn	S	P	Si	Ni	Cr	Fe
304	0.92	0.02	0.02	0.63	8.03	17.97	Bal.

**Table 2:** Mass-loss, corrosion-rate, and pitting parameters of 304 GW and 304 ZS after three years of Antarctic atmospheric exposure.

	304 GW	304 ZS
Mass loss after corrosion product removal	959 ± 140 mg	1175 ± 179 mg
Corrosion rate	1.428 μm y^−1^	1.643 μm y^−1^
Maximum pit depth	5.51 μm	8.24 μm
Average pit depth	4.16 ± 0.78 μm	5.85 ± 1.93 μm
Maximum pit width	43.95 μm	51.89 μm
Average pit width	27.09 ± 8.73 μm	31.69 ± 11.32 μm
Pit density	32.47 cm^−2^	43.31 cm^−2^

**Table 3:** Elemental compositions of the 304 stainless-steel substrates and passive films.

At. %	304 SS Substrate			Passive Film		
	304 Blank	304 GW	304 ZS	304 Blank	304 GW	304 ZS
Fe	70.13	73.21	73	45.71	57.78	46.93
O	3.4	2.92	0	32.7	13.84	32.56
Cr	19.01	17.69	19.83	14.43	21.96	13.78
Ni	7.47	6.18	7.17	7.16	6.42	6.72

**Table 4:** EIS fitting parameters for 304 Blank, 304 GW, and 304 ZS in 3.5 wt% NaCl solution.

Specimens	R_s_ (Ω cm^2^)	Q_dl_ (Ω^−1^ cm^−2^ s^n1^)	n_1_	R_ct_ (Ω cm^2^)	Q_D_ (Ω^−1^ cm^−2^ s^n2^)	n_2_
304 Blank	15.55	5.30 × 10^−5^	0.7868	143600	4.59 × 10^−5^	0.9475
304 GW	14.93	1.12 × 10^−4^	0.813	1386	2.05 × 10^−4^	0.875
304 ZS	14.55	6.45 × 10^−4^	0.531	254.9	5.00 × 10^−4^	0.802

**Table 5:** Calculated N_D_ and E_fb_ values of the passive films.

Specimens	N_D_ (cm^−3^)	E_fb_ (mV)
304 Blank	6.67 × 10^20^	−147.57
304 GW	3.35 × 10^21^	−420.56
304 ZS	5.97 × 10^21^	−567.31

## Data Availability

The original contributions presented in this study are included in the article/Supplementary Materials. Further inquiries can be directed to the corresponding authors.

## Supplementary Materials

The following supporting information can be downloaded at: https://www.mdpi.com/article/10.3390/ma19132754/s1, Figure S1. (a) Photograph of 304 Blank and (b,c) SEM morphologies of 304 Blank. Figure S2. EDS of the corrosion pit of 304 GW. Figure S3. EDS of the corrosion pit of 304 ZS. Figure S4. 304 Blank: (a) White-light interferometric images and (b) three-dimensional morphologies.
