# Supplementary material for: Corrosion Behavior of 304 Stainless Steel During Three-Year Atmospheric Field Exposure in Antarctica
Source: Materials (Basel). 2026 Jun 29;19(13):2754. doi: 10.3390/ma19132754 (PMC13362558; doi:10.3390/ma19132754)
Supplement: Supplementary file 1 [file materials-19-02754-s001.zip › materials-4373904-supplementary.pdf]

Supplementary Material

Corrosion Behavior of 304 Stainless Steel During Three-Year Atmospheric Field Exposure in Antarctica

Ting Peng [1], Shicheng Wang [4], Sizhi Zuo-Jiang [1], Zihao Tian [1], Yijing Sun [2,*], Xuzhou Jiang [1,3,*] and Dongbai Sun [1,*]

[1] School of Materials Science and Engineering & Southern Marine Science and Engineering Guangdong Laboratory (Zhuhai), Sun Yat-sen University, Guangzhou 510006, China
[2] Sino-French Institute of Nuclear Engineering, Sun Yat-sen University, Zhuhai 519082, China
[3] Nanotechnology Research Center, Sun Yat-sen University, Guangzhou 510275, China
[4] Guangzhou Customs Technology Center, Guangzhou 510000, China
* Correspondence: sunyij5@mail.sysu.edu.cn (Y.S.); jiangxzh7@mail.sysu.edu.cn (X.J.); sundongbai@mail.sysu.edu.cn (D.S.)

Supplementary Characterization

A pre-exposure elemental analysis was performed on the as-received coupons using a porta-ble X-ray fluorescence spectrometer (TRACER 5i, Bruker Corporation, Billerica, MA, USA). Macroscopic corrosion morphology was recorded using a digital camera (Sony Alpha 7C, Sony Corporation, Tokyo, Japan).The microscopic morphology was characterized by scanning electron microscopy（Axia ChemiSEM HiVac, Thermo Fisher Scientific, Waltham, MA, USA) and the elemental composition was shown by an energy dispersive spectrometer (SEM, EVO MA10 (W)). Surface images, three-dimensional surface morphologies, and cross-sectional profiles were acquired using a three-dimensional optical profilometer (ContourX-200, Bruker Scientific LLC, Billerica, MA, USA).

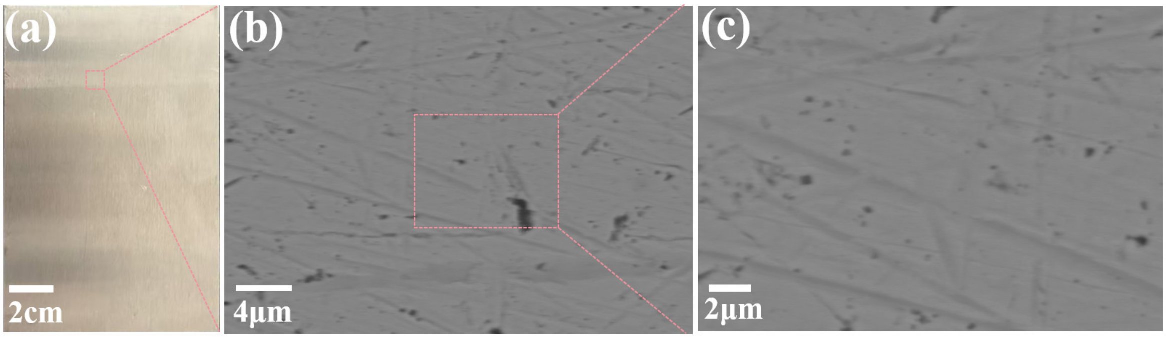

Figure S1. (a) Photograph of 304 Blank and (b,c) SEM morphologies of 304 Blank.

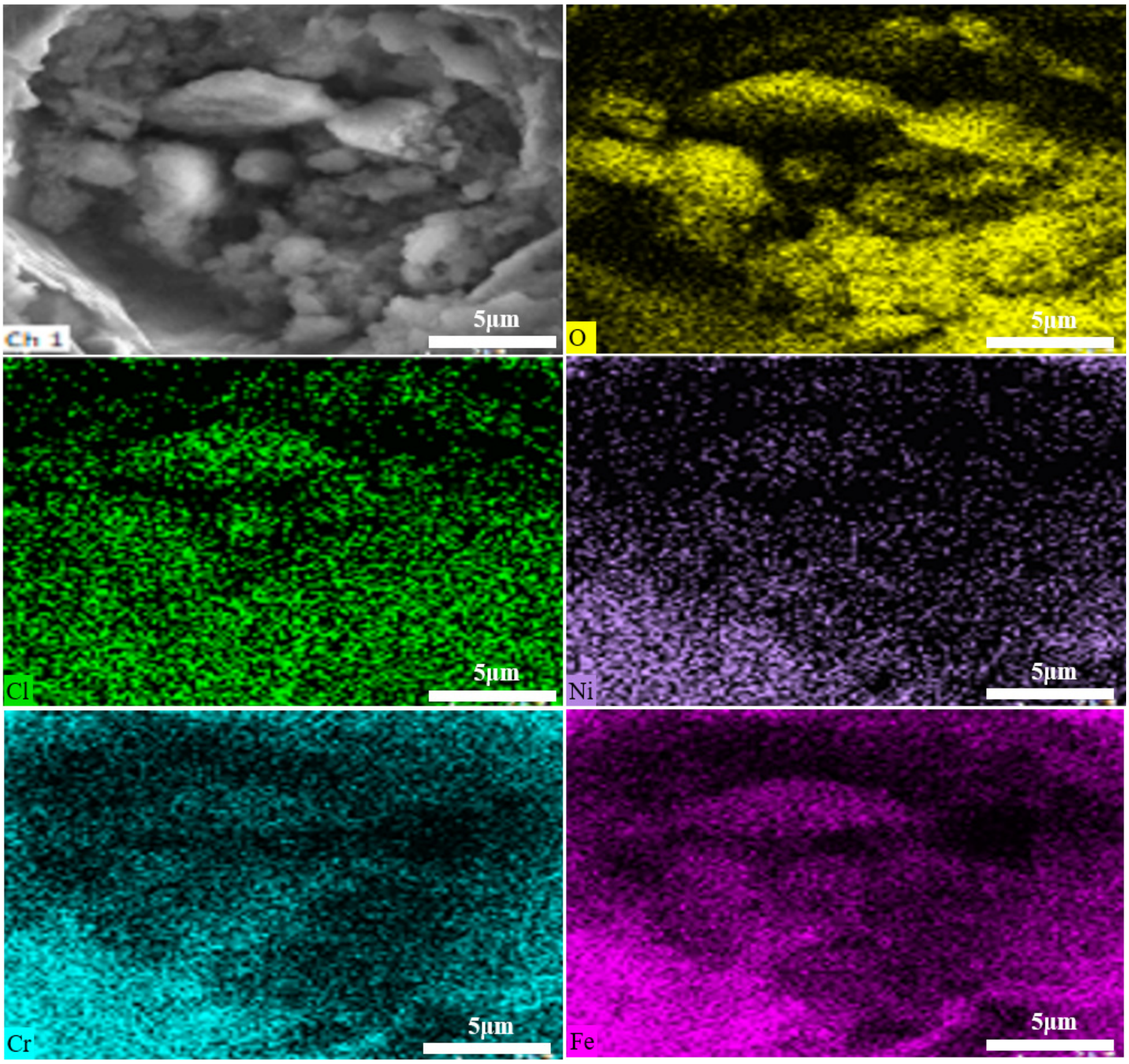

Figure S2. EDS of the corrosion pit of 304 GW.

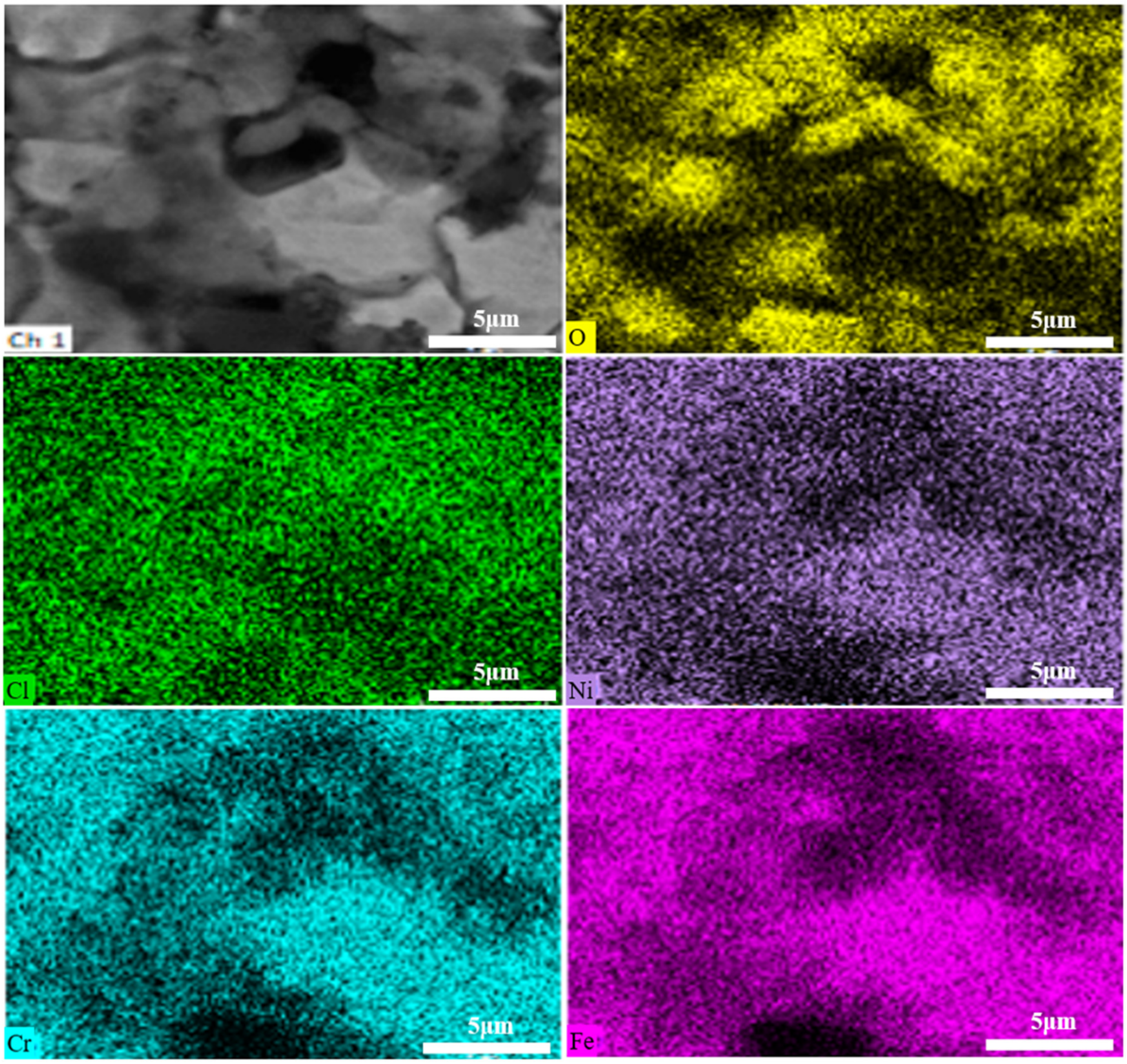

Figure S3. EDS of the corrosion pit of 304 ZS.

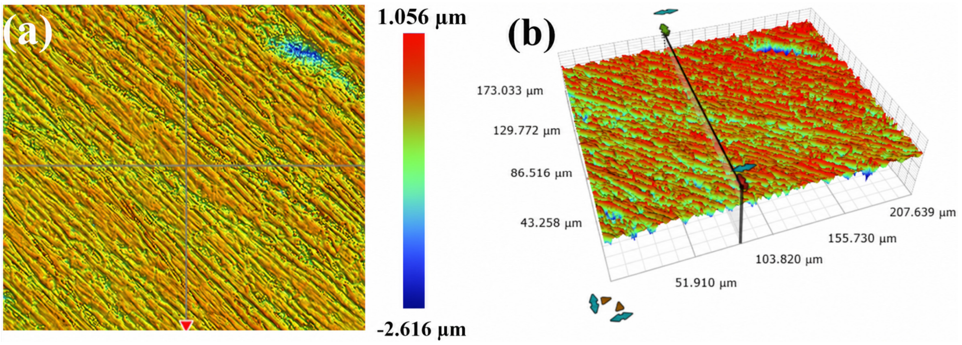

Figure S4. 304 Blank: (a)White-light interferometric images and (b)three-dimensional morphologies.

Disclaimer/Publisher's Note: The statements, opinions and data contained in all publications are solely those of the individual author(s) and contributor(s) and not of MDPI and/or the editor(s). MDPI and/or the editor(s) disclaim responsibility for any injury to people or property resulting from any ideas, methods, instructions or products referred to in the content.
